# Machine learning-based solution reveals cuproptosis features in inflammatory bowel disease

**DOI:** 10.3389/fimmu.2023.1136991

**Published:** 2023-05-18

**Authors:** Le Liu, Liping Liang, Chenghai Yang, Ye Chen

**Affiliations:** ^1^ Integrated Clinical Microecology Center, Shenzhen Hospital, Southern Medical University, Shenzhen, China; ^2^ Department of Gastroenterology, Nanfang Hospital, Southern Medical University, Guangzhou, China; ^3^ Guangdong Provincial Key Laboratory of Gastroenterology, Nanfang Hospital, Southern Medical University, Guangzhou, China

**Keywords:** inflammatory bowel disease, cuproptosis, immune landscape, single-cell RNA-seq, cluster

## Abstract

**Background:**

Cuproptosis, a new cell death mode, is majorly modulated by mitochondrial metabolism and protein lipoylation. Nonetheless, cuproptosis-related genes (CRGs) have not yet been thoroughly studied for their clinical significance and relationship with the immune microenvironment in inflammatory bowel disease (IBD).

**Methods:**

We screened CRGs that had a significant correlation with immune status, which was determined utilizing single-sample GSEA (ssGSEA) and Gene Expression Omnibus datasets (GSE75214). Furthermore, utilizing the R package “CensusClusterPlus”, these CRGs’ expression was used to obtain different patient clusters. Subsequently, gene-set enrichment analysis (GSEA), gene set variation analysis (GSVA), and CIBERSORT assessed the variations in the enrichment of gene function and the abundance of immune cell infiltration and immune functions across these clusters. Additionally, weighted gene co-expression network analysis (WGCNA) and analysis of differentially expressed genes (DEGs) were executed, and for the purpose of identifying hub genes between these clusters, the construction of protein-protein interaction (PPI) network was done. Lastly, we used the GSE36807 and GSE10616 datasets as external validation cohorts to validate the immune profiles linked to the expression of CRG. ScRNA-seq profiling was then carried out using the publicly available dataset to examine the CRGs expression in various cell clusters and under various conditions.

**Results:**

Three CRGs, PDHA1, DLD, and FDX1, had a significant association with different immune profiles in IBD. Patients were subsequently classified into two clusters: low expression levels of DLD and PDHA1, and high expression levels of FDX1 were observed in Cluster 1 compared to Cluster 2. According to GSEA, Cluster 2 had a close association with the RNA processes and protein synthesis whereas Cluster 1 was substantially linked to environmental stress response and metabolism regulations. Furthermore, Cluster 2 had more immune cell types, which were characterized by abundant memory B cells, CD4+ T memory activated cells, and follicular helper T cells, and higher levels of immune-related molecules (CD44, CD276,CTLA4 and ICOS) than Cluster 1. During the analysis, the PPI network was divided into three significant MCODEs using the Molecular Complex Detection (MCODE) algorithm. The three MCODEs containing four genes respectively were linked to mitochondrial metabolism, cell development, ion and amino acid transport. Finally, external validation cohorts validated these findings, and scRNA-seq profiling demonstrated diverse intestinal cellular compositions with a wide variation in CRGs expression in the gut of IBD patients.

**Conclusions:**

Cuproptosis has been implicated in IBD, with PDHA1, DLD, and FDX1 having the potential as immune biomarkers and therapeutic targets. These results offer a better understanding of the development of precise, dependable, and cutting-edge diagnosis and treatment of IBD.

## Introduction

Inflammatory Bowel Disease (IBD) is a chronic, recurrent intestinal condition with unknown mechanism and aetiology. It consists primarily of ulcerative colitis (UC) and Crohn’s disease (CD), which are both characterised by numerous intestinal ulcers. The active period alternates with the remission period, and various complications and extrenteral manifestations may occur. Including intestinal obstruction, intestinal perforation, fistula, abdominal abscess, perianal lesions, skin mucosal lesions, joint damage and liver lesions, high disability rate, most patients need lifelong treatment, seriously affecting the quality of life and survival of patients. IBD has long been considered a threat to public health in Western countries, but the increasing incidence of IBD in developing countries has made the disease a global problem in recent decades (1, 2). Due to the increasing number of patients, prolonged course of disease and high cost of treatment, the clinical and basic research of IBD has attracted more and more attention and gradually become a research hotspot in the field of gastrointestinal diseases. Many studies have found that genetic susceptibility, environmental factors, intestinal microecological changes, intestinal mucosal immune abnormalities and other factors are involved in the onset and progression of IBD (3). The improvement of living environment and sanitary conditions, the popularization of clinical antibiotics and the westernization of dietary habits are important environmental risk factors for the incidence of IBD in developing countries. However, there are still challenges in the diagnosis, prognosis, and tiered therapy of IBD. Moreover, there is a paucity of validated disease-indicative biomarkers (4–6).

Recently, metalloallostery has been used to describe the regulatory role of copper (Cu) in various cellular processes (7, 8). The term ‘metalloallostery’ describes Cu’s capacity to bind to newly undiscovered locations in proteins and regulate their activity. Thus, the current understanding of Cu has changed from that of a static co-factor to a regulatory factor. Increasing evidence suggests that Cu also acts as a dynamic signaling constituent exerting significant effects on varying activities, such as brain activity, cellular proliferation, autophagy, and lipolysis. Thus, regulating the biological availability of Cu both inside and outside of the cell is crucial for homeostasis (9, 10). Cuproptosis is defined as a non-apoptotic cell death process, wherein Cu binds directly to the tricarboxylic acid (TCA) cycle-related lipoylated components (11, 12). The accumulation of these Cu-bound, lipoylated mitochondrial proteins followed by the Fe-S cluster proteins’ loss causes proteotoxic stress and a different type of cell death. Various cell death types, including apoptosis, necroptosis, pyroptosis, and ferroptosis, have been studied in depth; however, Cu-induced cellular toxicity has received less attention. Several hypotheses have been presented to explain the mechanism of Cu-induced cell death, such as apoptosis activation, cell death independent of caspase, the synthesis of ROS, and the suppression of the ubiquitin-proteasome system. Cu metalloallostery is the binding of Cu to the non-catalytic areas of proteins to regulate pathways like proliferation, lipolysis, and autophagy. Nonetheless, non-specific Cu binding has often been reported as a probable process of Cu toxicity and is frequently used to criticize metalloallostery. Wilson disease hepatocytes, which hyper-accumulate Cu due to ATP7B mutations, produce less lipoylated protein and iron-sulfur clusters, indicating the potential role of cuproptosis in disease pathogenesis. To remove damaged mitochondria and presumably reduce cuproptosis, Cu metalloallostery is activated in Wilson disease hepatocytes to elevate autophagy and lysosome biogenesis in a direct manner. Various studies confirmed the existence of numerous extra protein binding sites for exchangeable Cu. Under normal circumstances, Cu binding to mitochondria’s lipoylated proteins is an exciting potential revealed by the discovery of cuproptosis (12, 13). Cu obtainability might constitute a temporary allosteric control process mediated by protein aggregation on smaller scales. Moreover, Cu overload caused by ionophores or illnesses can overwhelm this highly calibrated system, resulting in Cu-mediated aggregation and cell death. The discovery of cuproptosis has paved the way for further research into the unique elements of mitochondrial biology in autoimmune disorders and normal cellular function.

IBD has become a global health concern, and its complexity creates significant obstacles for patients, researchers, and caregivers. Despite the fact that many efforts have been made to overcome these obstacles and produce viable treatments, the progress made thus far is insufficient. Throughout the past few decades, various forms of cell signaling blocking medicines, including biologics, have improved clinical response and clinical remission rates for IBD. In patients with CD or UC, the efficacy of the majority of treatments has a biological upper limit. Hence, there is an urgent need to create new research avenues and breakthrough IBD treatments in order to overcome the efficacy ceiling. Other diseases (such as atherosclerosis) have been thoroughly researched in terms of genomic, transcriptome, epigenome, and proteome alterations, however information from the omics data sets of patients with IBD is limited. Data from all omics research contribute to the analysis of the pathobiological mechanisms of IBD, although the relative value of various omics components may vary. It is possible that distinct pathophysiological variables may predominate in distinct subgroups of IBD patients. Cu is an essential trace element that acts as a co-factor for enzymes in environments where cells are exposed to correct quantities of the metal, which can be poisonous to the cells if present in unsuitable amounts. Interestingly, the metabolic imbalance produced by copper has a high correlation with the development of IBD, and the copper-to-zinc ratio has a substantial correlation with CRP and calprotectin in people who have active IBD (14). A regulator of the Cu transport pathway, COMMD1 has been demonstrated to suppress NF-κB activation (15, 16). Eliminating COMMD1 in myeloid cells has been linked to more severe inflammatory responses, suggesting that sustained COMMD1 inhibition may be unfavorable throughout chronic inflammation. As a novel pattern of cell death, cuproptosis has garnered considerable interest. When the mitochondrial respiration chain is disturbed, excess copper binds directly to the lipoylated components of the TCA cycle. In addition, cell death is intimately linked to gut barrier degradation and anti-inflammatory cell inhibition of IBD, such as in goblet cells and Tregs. Thus, it is plausible to hypothesise that cuproptosis may have an impact on the onset of IBD. However, the mechanism is poorly known, and few studies have employed bioinformatics to investigate the involvement of cuproptosis-related genes (CRGs) and related intestinal cell clusters in IBD, which could lead to a new line of enquiry into the disease. The current study aims to investigate the role of CRGs in the aetiology of IBD and immunological regulation, as well as to identify possible cuproptosis-associated candidate biomarkers and therapeutic targets.

## Methods and materials

### Data sources and processing

The “GEOquery” R package (version 2.66.0) was used to get three microarray datasets (GSE75214, GSE36807, and GSE10616) linked to IBD from the Gene Expression Omnibus (GEO) database, which is located at https://www.ncbi.nlm.nih.gov/geo/. The GSE75214 dataset including 22 healthy, 75 CD and 97 UC samples were selected as training set. Both the GSE36807 dataset, which had tissues from 7 healthy samples, 13 CD samples, and 15 UC samples, and the GSE10616 dataset, which contained tissues from 16 healthy samples, 32 CD samples, and 10 UC samples, were chosen for an external validation analysis. The gene symbol conversion for each dataset was annotated using the corresponding platform file.

### Consensus clustering

We used Spearman’s coefficient for investigating the link between the expression of CRGs and the findings of the ssGSEA analysis to determine the effect of cuproptosis on the immunological profiles of IBD. As FDX1, DLD, and PDHA1 possessed the strongest correlation with immune signature based on the mean value of correlation and the median value of P-value, these three genes were executed consensus clustering on IBD sample data. Utilizing the R package “ConsensusClusterPlus” consensus clustering and visualization of the results were performed (17). Additionally, we employed the R package “FactoMineR” to test the effectiveness of the aforementioned consensus clustering via principal component analysis (PCA).

### Gene set enrichment analysis

Utilizing the default defined set of genes, GSEA software (https://www.gsea-msigdb.org/gsea/) was employed for determining the enrichment of distinct pathways in the two clusters (18). As the pre-defined ontology gene set, “c2.cp.kegg.v7.4.symbols.gmt” was chosen from the MSigDB Collection and regarded a pathway as a substantially enriched pathway with the absolute normalized enrichment score that was greater than one (|NES| >1) and adjusted p-value that was less than 0.05.

### Establishment and evaluation of the nomogram

Nomograms can include multiple different factors that influence prognosis simultaneously to predict the study cohort’s survival or occurrence (19). Construction of a predictive nomogram based on the aforementioned characteristics (FDX1, DLD, PDHA1) was done utilizing the “rms” R software (version 6.5.0) (20). In order to make a comparison between the expected values and the standard values, calibration curves were utilised. The decision curve analysis (DCA) approach was applied so that the nomogram model’s ability to forecast could be evaluated. For the purpose of visualising the receiver operating characteristic (ROC) curves, the “pROC” R package was utilised.

### Evaluating immune cell infiltrations

Using gene expression profiles, the CIBERSORT approach, which excels at reducing noise and recognizing related cell types, was applied for identifying the tissues’ immune cell composition. For each sample, CIBERSORT employs Monte Carlo sampling to calculate the inverse fold product p-value. Only samples with p-values less than 0.05 were deemed accurate immune cell fractions. The sum of the proportions of the 22 immune cells in each sample was one (21). Using ssGSEA, a GSEA extension, separate enrichment scores were generated for each pairing of a gene set and sample (22).

### DEGs and WGCNA analysis

We sequentially analyzed DEGs and WGCNA to identify the hub genes that assisted the biological divergences between various subclusters. First, the ‘limma’ R package was used to contrast transcriptome data (FPKM normalization) and identify DEGs. The |log2FoldChange| > 1 and adjusted p-value < 0.05 served as screening thresholds, and a heat map and a volcano plot were utilized to represent the findings. Following that, we used the “WGCNA” R package to run WGCNA (software = 12) on the DEGs, which organises strongly linked genes into modules and assesses the connection between modules and external sample attributes. The relationships between clustering and immune checkpoints, and ssGSEA characteristics were investigated. Lastly, the module of genes closely associated with clustering (greenyellow module) was selected for subsequent analyses.

### Analysis of functional enrichment and protein-protein interaction network

The gene list was uploaded to Metascape (http://metascape.org/) in order to undertake pathway and process enrichment analysis as well as PPI enrichment analysis. This was done in order to further investigate the aforementioned module of genes. During the functional enrichment study, ontology sources from GO Biological Processes, Canonical Pathways, KEGG Pathway, Reactome Gene Sets, and WikiPathways were utilised. Additionally, a PPI enrichment analysis was conducted. The Molecular Complex Detection (MCODE) algorithm was utilized to separate proteins and construct interaction networks if the network’s protein number was between 3 and 500 (23, 24).

### Gene set variation analysis

It is feasible to discover how the enriched gene sets of the various clusters are distinct from one another by utilising GSVA, which is a method of nonparametric, unsupervised analysis. In order to evaluate the biological roles played by the gene sets, the “GSVA” package version 1.46.0 in R was utilised to assign signalling pathway variation scores to each of the gene sets. Gene sets were collected from the Molecular Signatures Database, which can be found at http://software.broad-institute.org/gsea/msigdb. The definition of a significant change was a |t value of GSVA score| that was more than 1.

### ScRNA-seq data processing and identification of cell types

The single cell data set of colon tissue used in this study was come from a previous study, which contained three conditions (healthy, non-inflammation and inflammation) (25). Using the “Seurat” package, the processed data was examined. Scrublet was used to find doublets and get rid of low-quality cells (26). The expression matrix was standardised using the log2 (CPM+1) values as the input matrix for the subsequent analysis. Principal component analysis was carried out after high variable genes were found using the “FindVariableGenes” function. Based on the uniform manifold approximation and projection (UMAP) embedding of the initial work, the dimension reduction of single-cell visualisation was carried out. Then, the “DimPlot” function was used to visualise the single-cell UMAP plot, while the “FeaturePlot” and “Dotplot” functions were used to visualise the hub gene expression plot. On the basis of the expression of known marker genes, cell clusters from each location were manually categorised into three compartments: Epithelial (EPCAM, KRT8, and KRT18), Stromal (CDH5, COL1A1, COL1A2, COL6A2, and VWF), and Immune (CD45/PTPRC, CD3D, CD3G, CD3E, CD79A, CD79B, CD14, CD16, CD68, CD83, CSF1R, FCER1G). Subtype annotation of each main compartment in the original study was used for our study.

## Results

### Identifying CRGs linked to IBD immune profiles

**Figure 1:**
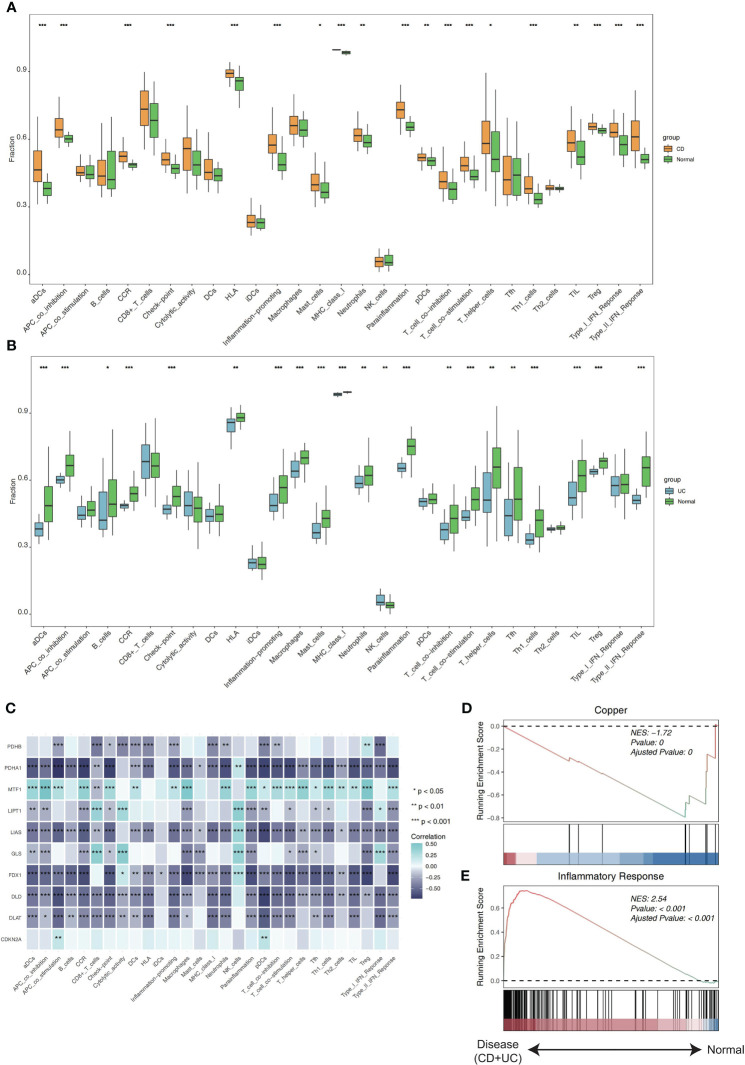
The correlation of CRGs with ssGSEA in the GSE75214 cohort. **(A)** Boxplots of the differentially infiltrated immune cells between CD and normal groups. **(B)** Boxplots of the differentially infiltrated immune cells between UC and normal groups. **(C)** The correlation of CRGs with ssGSEA. **(D)** The GSEA plot of the cuproptosis-related gene sets. **(E)** The GSEA plot of the inflammatory response-related gene sets. *p< 0.05, **p< 0.01, ***p< 0.001.

To investigate if the CRGs’ expression affected IBD immune profiles, we extracted the expression of 10 CRGs from previous study (27) and used ssGSEA to estimate the immune cell infiltration of 194 samples. The clustering was done for the included samples utilizing the average linkage hierarchical clustering approach using relative immune cell abundance, and the normalized enrichment score of immune infiltrates is presented in the heatmap (**Supplementary Figures S1A, B**). Compared to healthy people, CD patients showed increased levels of aDCs, pDCs, mast cells, neutrophils, T helper cells, Th1 cells, TIL, and Treg, according to the results of a differential study of immune cell infiltration. Also, CD patients had significantly greater levels of immune function subtypes like APC co-inhibition, CCR, Check-point, HLA, Inflammation-promoting, MHC_class_I, Parainflammation, T_cell co-inhibition, T_cell co-stimulation, Type_I_IFN_Reponse, Type_II_IFN_Reponse (**Figure 1A**). Compared to healthy people, UC patients had reduced levels of aDCs, macrophages, mast cells, neutrophils, T helper cells, Tfh, Th1 cells, TIL, and Treg. Moreover, APC co-inhibition, CCR, Check-point, HLA, Inflammation-promoting, MHC_class_I, Parainflammation, T_cell co-inhibition, T_cell co-stimulation, Type_II_IFN_Reponse were all shown to be significantly lower in UC patients (**Figure 1B**). Using Spearman correlation analysis to examine the relationship between immune cell subtypes/immune function subtypes and the expression of 10 CRGs, the findings indicated that MTF1 was significantly positively correlated with nearly all immune cell subtypes and immune function subtypes, whereas PDHB, PDHA1, LIAS, FDX1, DLD, and DLAT had a negative correlation (**Figure 1C**). Additionally, inflammatory response and cuproptosis gene sets, respectively, were used for the GSEA enrichment analysis. The outcomes demonstrated that the enrichment of cuproptosis differed greatly from an inflammatory response. Whereas inflammatory response was primarily enriched in the disease group, cuproptosis was primarily enriched in the normal group (**Figures 1D, E**). The result suggested that cuproptosis in IBD may be a better indicator than other inflammatory pathways.

### IBD patients’ consensus clustering using PDHA1, DLD, and FDX1

**Figure 2:**
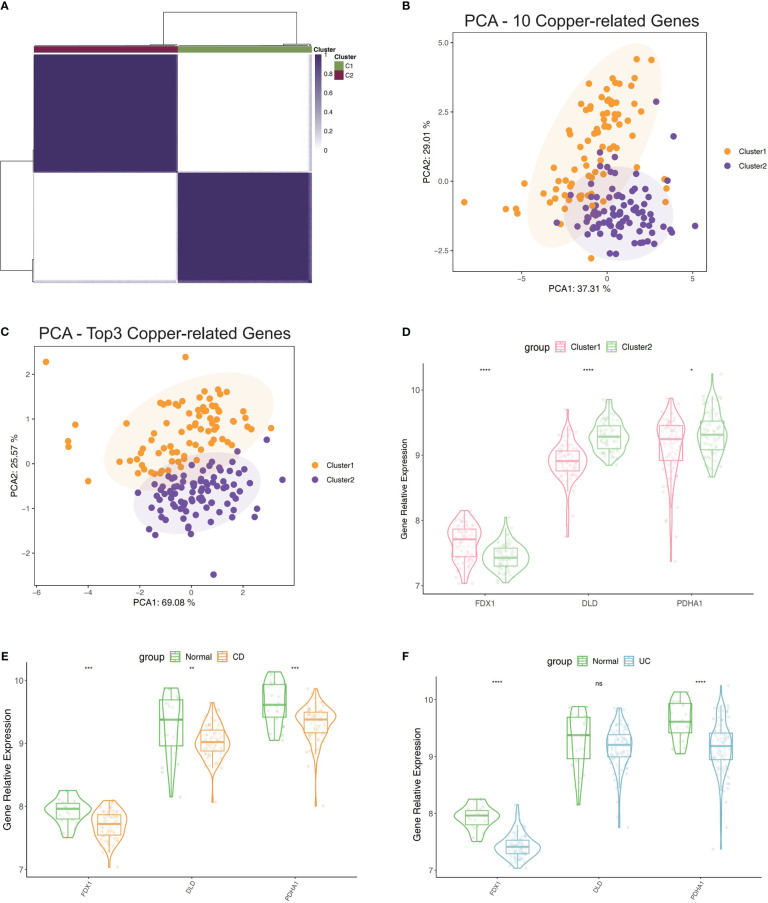
Clustering of IBD patients in the GSE75214 dataset using the expression of PDHA1, DLD, and FDX1. **(A)** Matrix of consensus clustering for k = 2. **(B)** The findings of clustering-related PCA using 10 CRGs. **(C)** The findings of PCA of clustering using top3 CRGs. **(D)** Contrast of the top3 CRGs’ expression levels between Cluster 1 and 2. **(E)** Contrast of the top3 CRGs’ expression levels between CD samples and normal samples. **(F)** Contrast of the top3 CRGs’ expression levels between UC samples and normal samples. *p< 0.05, **p< 0.01, ***p< 0.001, ****p< 0.0001, ns, no significance.

The top 3 genes (PDHA1, DLD, and FDX1) were chosen for further clustering analysis after the correlation mean value and median value of P values of all immune score related to each gene were obtained (**Table S1**). Then the extraction of expression data of PDHA1, DLD, and FDX1 in the included samples was done, and we also carried out the consensus clustering in GSE75214 to establish two clusters of patients (**Figure 2A**). The PCA plot showed that the clustering described above had good distinction efficiency compared with clustering using all CRGs (**Figures 2B, C**). Additionally, we performed a similar clustering analysis using immune checkpoints-related genes and discovered that the outcomes were comparable to those obtained using cuproptosis-related genes, whereas the outcomes of the PCA analysis revealed that the top three cuproptosis-related markers had a much stronger differentiation than immune checkpoints (**Supplementary Figures S1C, D**). Cluster 1 had a lower expression of DLD and PDHA1 and a higher expression of FDX1 (**Figure 2D**) than Cluster 2. Additionally, these three regulators’ expression levels were contrasted between IBD and normal tissues, which revealed a lower expression in IBD tissues compared to normal tissues (**Figures 2E, F**).

### Development of a IBD diagnostic column line graph

**Figure 3:**
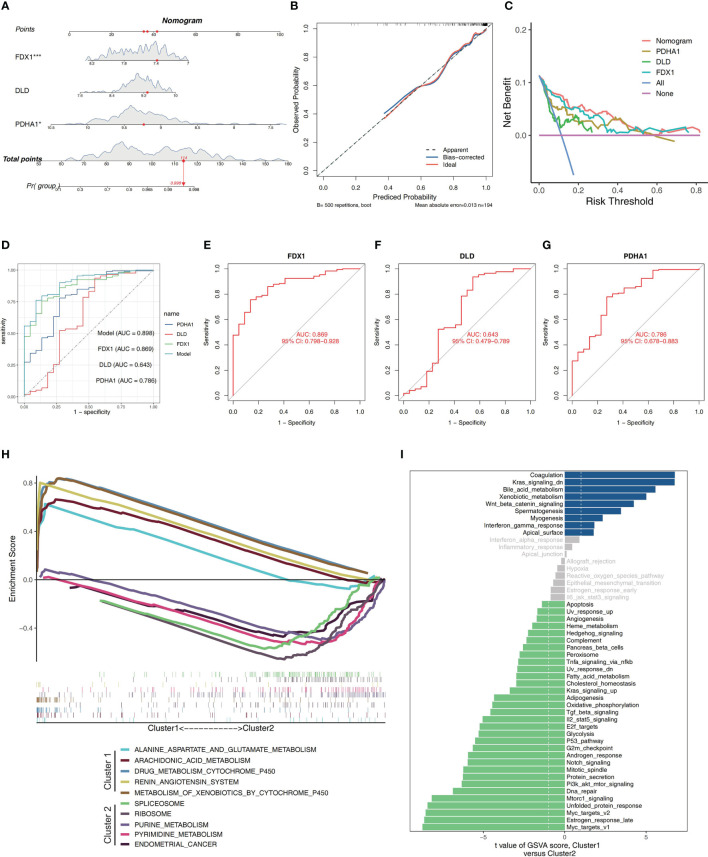
Construction of a nomogram and differences in GSEA between two clusters. **(A)** Nomogram showing three featured genes used in the diagnosis of patients with IBD. **(B)** Calibration curve showing predicted performance of the column line graph model. **(C)** The clinical benefits of the model are evaluated using DCA curves. **(D)** ROC curves are used to evaluate the clinical value of the model. **(E–G)** The ROC curves of the training set’s feature genes. **(H)** The biological pathways’ enrichment tendency between two clusters. **(I)** The bar plots illustrate the distribution of the t values of the GSVA scores generated for a variety of pathways.

As a diagnostic tool for IBD progression, a nomogram was constructed by incorporating the characteristic genes (**Figure 3A**). Each characteristic gene in the nomogram was assigned a score, and the sum of all the scores for the characteristic genes was used to determine the final score. The total score represented various IBD risks. According to the calibration curves, the results that the model had predicted were almost identical to the results that were actually obtained (**Figure 3B**). The curve for the “column line graph” is higher than the control line in the DCA, and the curve for the “FDX1, DLD, and PDHA1” suggests that patients may benefit from the column line graph model at a high-risk threshold of 0 to 1. A larger clinical benefit was obtained from the use of the column line graph model as opposed to the “FDX1, DLD, and PDHA1” curve (**Figure 3C**). The correctness of the model may also be confirmed using the ROC curve analysis, and the results implied that the clinical significance of the model to predict the occurrence probability of IBD was superior to that of a single independent predictive factor (**Figures 3D–G**). Based on these studies, it appears that the pathogenesis of IBD involves all main CRGs.

The pathway enrichment between the two clusters was contrasted utilizing GSEA. Biological pathways associated with RNA processes and protein synthesis, including SPLICEOSOME, and RIBOSOME, were enriched in Cluster 2. In Cluster 1, environmental stress response and metabolism-related biological pathways, such as ALANINE_ASPARTATE_AND_GLUTAMATE_METABOLISM, ARACHIDONIC_ACID_METABOLISM, DRUG_METABOLISM_CYTOCHROME_P450, RENIN_ANGIOTENSIN_SYSTEM and METABOLISM_OF_XENOBIOTICS_BY_CYTOCHROME_P450, were enriched (**Figure 3H**). The findings revealed that Cluster 1 had a close association with metabolism modulation in IBD. Additionally, the GSVA analysis suggested that these two clusters enriched in many different pathways, including Il2_stat5_signaling, Tnfa_signaling_via_nfkb, Tgf_beta_signaling, Interferon_gamma_response, Kras_signaling_up, G2m_checkpoint, Apoptosis, Dna_repair,Unfolded_protein_response, Myc_targets_v1, Mtorc1_signaling, Heme_metabolism, Oxidative_phosphorylation, Fatty_acid_metabolism, Bile_acid_metabolism, and so on, which were involved in immune responses, cell death and energy metabolism (**Figure 3I**).

### Association between clustering and immune cell infiltrations

**Figure 4:**
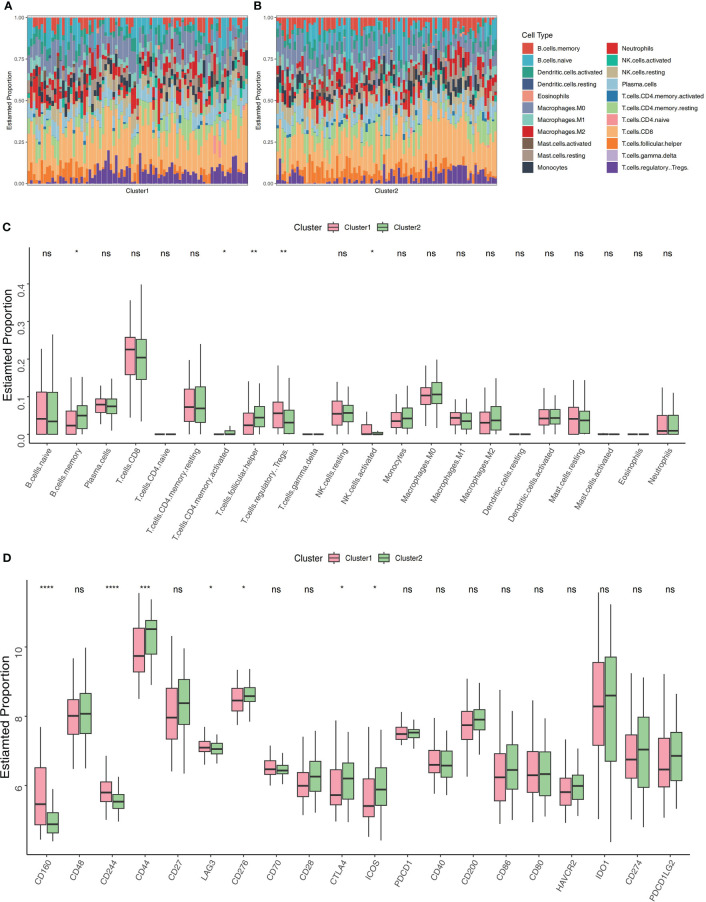
Profiling of immune infiltrations in the two Clusters. **(A)** Stacked bar chart illustrates the 22 immune cell types’ distribution in Cluster 1. **(B)** Stacked bar chart illustrates the 22 immune cell types’ distribution in Cluster 2. **(C)** Differential study of immune cell infiltrations in Cluster 1 and 2. **(D)** Cluster 1 versus Cluster 2 in terms of molecules associated to the immune. *p< 0.05, **p< 0.01, ***p< 0.001, ****p< 0.0001, ns, no significance.

A CIBERSORT analysis was performed, and a comparison of the abundance of immune-related cell subtypes was made. This was done so that we could examine the degree to which the two Clusters differed in the immune infiltration profiles they displayed. The findings indicated that there was a difference in the immune microenvironment between cuproptosis-related Cluster1 and Cluster2 (**Figures 4A, B**). CIBERSORT analysis revealed that memory B cells, CD4+ T memory activated cells, and follicular helper T cells had elevated percentages in Cluster 2 while Cluster 1 had a high expression of activated NK cells, and regulatory T cells. Thus, it’s possible that cuproptosis-related Cluster 2 may have a faster immune response capacity (**Figure 4C**). As immune modifiers and monoclonal antibodies have become more significant in the IBD treatment, we evaluated the critical immune-related biomarkers’ expression levels in the two clusters. Compared to Cluster 2, CD160, CD244 and LAG3 were elevated while CD44, CD276,CTLA4 and ICOS were decreased in Cluster1 (**Figure 4D**).

### Identifying key modules genes, functional enrichment and PPI network analysis

**Figure 5:**
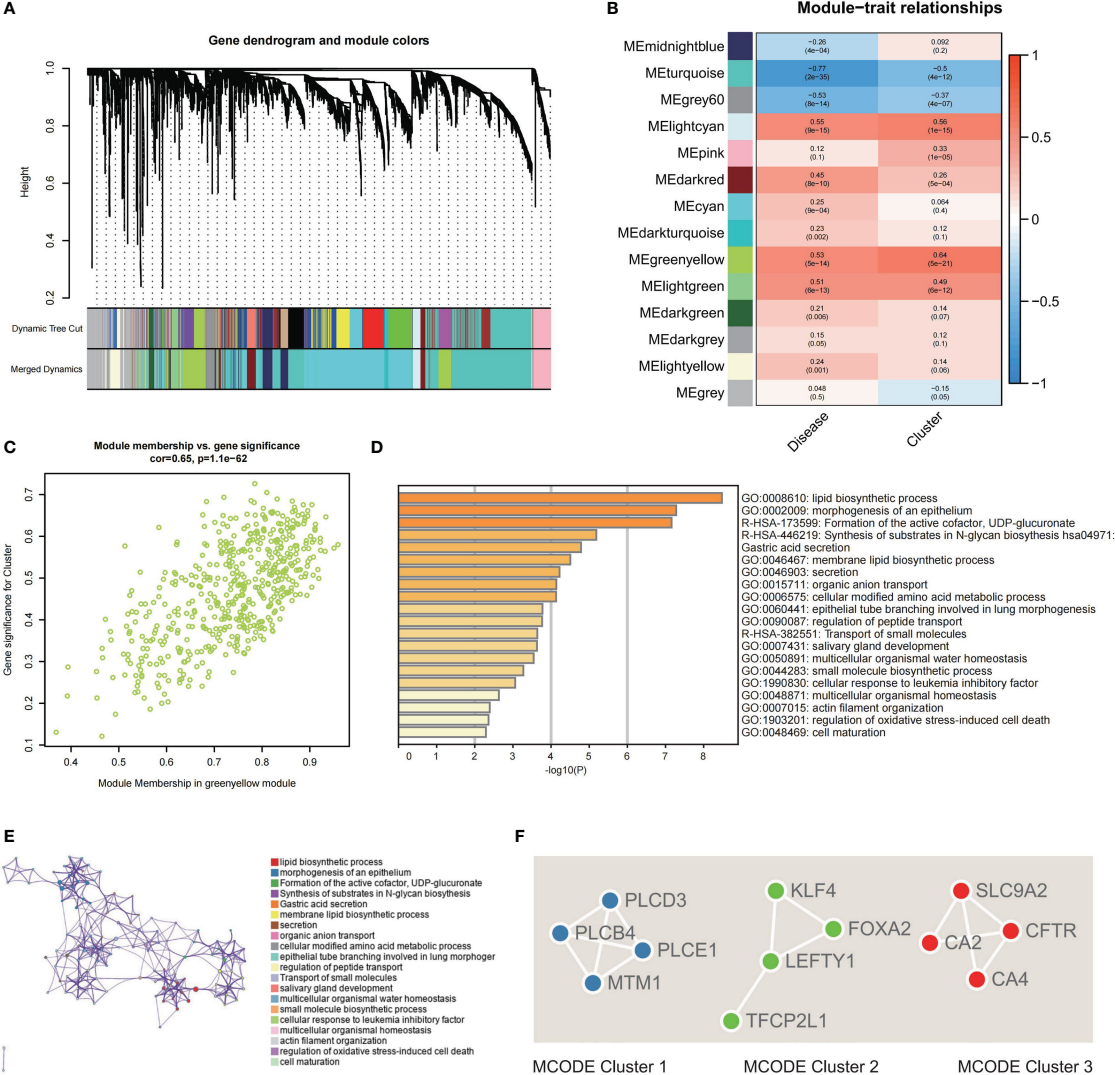
Screening of hub genes related to clustering and construction of a PPI network. **(A)** Dendrogram of 5000 genes containing 266 DEGs and 4734 top MAD-ordered genes clustered using a dissimilarity measure. **(B)** The correlation study between module eigengenes and disease state and clustering is depicted as a heatmap. Each column includes the matching correlation and p-value. **(C)** Scatter plot of module membership in greenyellow module versus the gene significance for Cluster. **(D)** Analysis of functional enrichment in different ontology sources. **(E)** Chosen enriched terms for a network, coloured according to the ID of the cluster group. **(F)** PPI network analysis for the three specified MCODE components.

The DEGs were identified between Cluster 1 and Cluster 2, revealing 266 DEGs (upregulated, 179; downregulated, 87; **Supplementary Figures S2C, D**). To improve the accuracy of WGCNA, we extracted the top 4734 genes having the greatest median absolute deviation **(**MAD) and combined the previous 266 DEGs. Lastly, a total of 5000 genes were used for WGCNA. The power of = 12 (scale-free R2 = 0.9) was chosen as the soft-thresholding value for constructing a scale-free network (**Supplementary Figures S2A, B**). Fourteen modules in total were discovered utilizing the average linkage hierarchical clustering (**Figure 5A**). Moreover, the module eigengenes (MEs) of the lightcyan module, and greenyellow module were observed to have a positive relationship (>0.5) with the clustering, and the MEs of the lightcyan, greenyellow, and lightgreen modules had a positive correlation with IBD (>0.5, **Figure 5B**). For further investigation, these modules were chosen as significant clinical modules. The greenyellow module had the highest overall correlation with the clustering and disease as determined by module connectivity and quantified by the absolute value of Pearson's correlation and clinical trait association (**Figure 5C**). Furthermore, the greenyellow module was utilized to detect hub genes.

For functional enrichment analysis, hub genes discovered by the greenyellow module were imported into Metascape to further investigate them, and the construction of the PPI network was done. In the functional enrichment analysis, the most substantial pathways were associated with metabolic regulation, such as lipid biosynthetic process, membrane lipid biosynthetic process, cellular modified amino acid metabolic process, and regulation of peptide transport (**Figures 5D, E**). Then, the MCODE algorithm classified the whole PPI network into three primary MCODEs in the PPI network analysis, each comprising four genes (**Supplementary Figure S3**). MCODE1 (PLCE1, PLCD3, PLCB4, and MTM1) was strongly correlated with metabolic process (**Figure 5F**). MCODE2 (TFCP2L1, LEFTY1, FOXA2, and KLF4) was strongly correlated with the cell maturation and development. MCODE3 (CFTR, CA2, SLC9A2, and CA4) was strongly correlated with the regulation of peptide and ion transport (**Figure 5F**).

### External validation of immune cell landscape in two clusters

**Figure 6:**
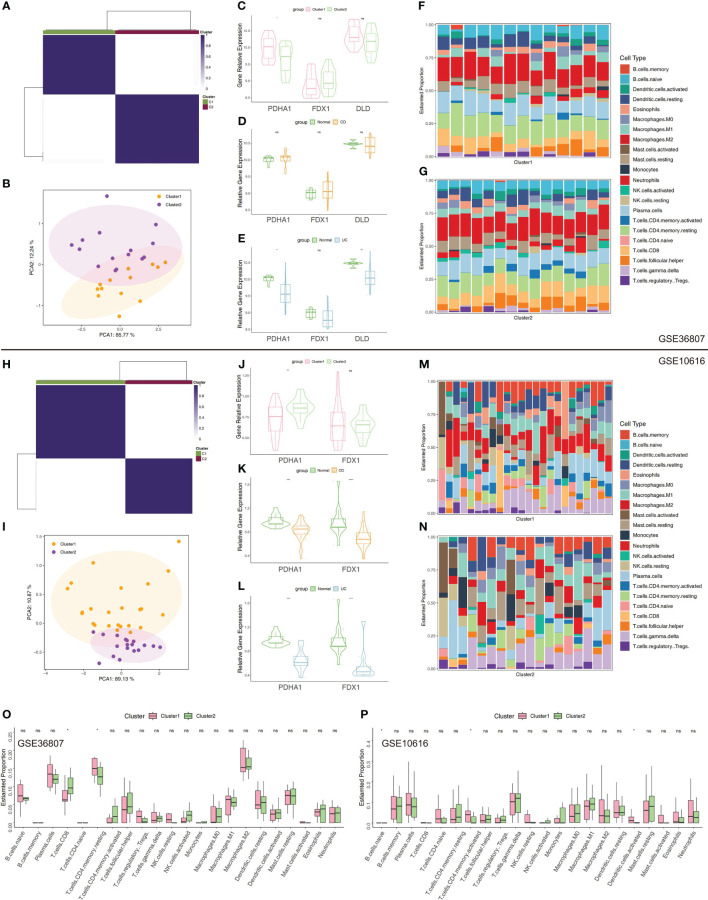
Validation of clustering using top3 CRGs in GSE36807 and GSE10616. **(A)** Matrix of consensus clustering for k = 2. **(B)** The PCA findings using top3 CRGs (FDX1, DLD, and PDHA1). **(C)** The expression levels of the chosen CRGs between Cluster 1 and 2. **(D)** The expression levels of the chosen CRGs between CD samples and normal samples. **(E)** The expression levels of the chosen CRGs between UC samples and normal samples. **(F)** Stacked bar chart illustrates the 22 immune cell types’ distribution in Cluster 1. **(G)** Stacked bar chart illustrates the 22 immune cell types’ distribution in Cluster 2. **(H)** Matrix of consensus clustering for k = 2. **(I)** The PCA findings using top3 CRGs. **(J)** The expression levels of the chosen CRGs between Cluster 1 and Cluster 2 (“DLD” was not detected in GSE10616). **(K)** The expression levels of the chosen CRGs between CD samples and normal samples. **(L)** The expression levels of the chosen CRGs between UC samples and normal samples. **(M)** Stacked bar chart illustrates the 22 immune cell types’ distribution in Cluster 1. **(N)** Stacked bar chart illustrates the 22 immune cell types’ distribution in Cluster 2. **(O, P)** Immune cell infiltration-related differential analysis in Cluster 1 and 2. *p< 0.05, **p< 0.01, ***p< 0.001, ****p< 0.0001, ns, no significance.

The clustering based on three CRGs (PDHA1, DLD, and FDX1) was externally validated utilizing the GSE36807 and GSE10616 datasets. Consensus clustering was performed in GSE36807, which divided the samples into two clusters (**Figure 6A**). The PCA plot showed that the clustering described above has a high degree of distinction efficiency (**Figure 6B**). Cluster 1 comprised 13 samples whereas Cluster 2 comprised 15 samples, and the latter had substantially higher FDX1 expression levels (**Figure 6C**). Moreover, patients with UC had significantly lower PDHA1 and DLD expression levels compared to normal controls (**Figures 6D, E**). Furthermore, CIBERSORT revealed that contrasted to Cluster 1, substantially higher CD8 T cells and less CD4 T memory resting cells in Cluster 2 (**Figures 6F, G, O**). Thus, these findings revealed that the top3 CRGs-based patients clustering exhibited different immune profiles, which were similarly verified in data set GEO10616 (**Figures 6H–N, P**).

### ScRNA-seq analysis for the top CRGs

**Figure 7:**
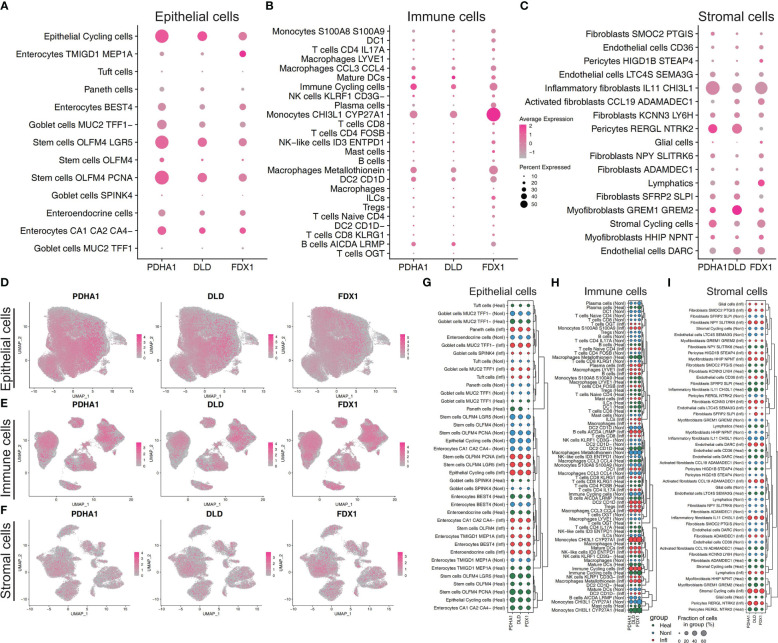
Cuproptosis-related features in scRNA-seq profiling. Dot plot showing the relative expression of three cuproptosis-related hub genes from epithelial cell subtypes **(A)**, immune cell subtypes **(B)**, and stromal cell subtypes **(C)**. Feature plot showing the normalized gene expression of three hub genes colored by epithelial cell subtypes **(D)**, immune cell subtypes **(E)**, and stromal cell subtypes **(F)**. Dot plot showing the relative expression of three cuproptosis-related hub genes across three conditions (normal, non-inflammation, and inflammation) from epithelial cell subtypes **(G)**, immune cell subtypes **(H)**, and stromal cell subtypes **(I)**. According to the similarity of gene expression in different cell subtypes, hierarchical clustering is conducted to highlight the specific gene expression patterns of genes in different subtypes.

Follow the method used by Xavier RJ et al., annotation by cell-type markers first broadly classified cells into three major cell-type compartments: 97,345 epithelial cells, 39,424 stromal cells, and 142,045 immune cells. The epithelial cells can be further divided into 13 cell subtypes, including Enterocytes, Enteroendocrine cells, Epithelial Cycling cells, Goblet cells, Paneth cells, Stem cells and Tuft cells. The immune cells can be further divided into 25 cell subtypes, including B cells, DC, ILCs, Immune Cycling cells, Macrophages, Mast cells, Mature DCs, Monocytes, NK, Plasma, CD4+ T cells, CD8+ cells, Naïve T cells, and Treg. The stromal cells can be further divided into 17 cell subtypes, including Activated fibroblasts, Endothelial cells, Fibroblasts, Glial cells, Inflammatory fibroblasts, Lymphatics, Myofibroblasts, Pericytes, and Stromal Cycling cells (25). The percentage of expression of cuproptosis-related hub genes in each compartment was shown in dot plots and UMAP plots (**Figures 7A–F**). We can find these three hub genes (PDHA1, DLD, FDX1) were highly expressed in Epithelial Cycling cells, Stem cells OLFM4 LGR5, Stem cells OLFM4 PCNA from Epithelial cells, which indicated they may play an important role in the regulation of epithelial cells proliferation. For immune cell subtypes, the FDX1 was highly expressed in the Monocytes (CHI3L1, CYP27A1) compared with other hub genes, which indicated this gene may play a key role in the activation and recruitment of monocytes. For stromal cells, we can find these three genes mainly located in the Inflammatory fibroblasts (IL11, CHI3L1), Myofibroblasts (GREM1, GREM2) and Pericytes cells. Furthermore, we also explored the relative expression differences of these cuproptosis-related hub genes under different conditions. We can find that these genes have specific differences in different cell subtypes and under different conditions, which highlights the heterogeneity of each cell subtype and the specific expression patterns of these genes in different cell subtypes (**Figures 7G–I**). In addition, we can find that these genes are mainly highly expressed in epithelial and immune cell subtypes, compared with stromal cells which illustrated that these three genes mainly played a potential role in the transcription and regulation of epithelial and immune cell subtypes (**Figures 7G–I**).

## Discussion

Multiple forms of cell death have been identified to date, including cuproptosis, pyroptosis, necroptosis, ferroptosis, autophagy, and apoptosis. Pyroptosis, apoptosis, and necroptosis induce instability of the membrane and rupture of the cell via distinct molecular and cellular processes, including ionic gradients and inflammatory caspases (28). Autophagy results in the disintegration of organelles, which supply metabolites, prevent DNA damage, and resist oxidative stress (29). Ferroptosis is a type of oxidative cell death dependent on iron owing to unrestrained lipid peroxidation (30). Each cell death type signifies a distinct process as well as illustrates an immune response under various settings. The significance of these processes resides in the discovery of new targets with potential effectiveness and implementation feasibility. Cuproptosis is a novel mechanism of cell death caused by an excess of Cu and succeeding disruption to the TCA pathways in the mitochondria (31, 32). Despite the significant breakthroughs in the numerous forms of controlled cell death, the processes and implications of cuproptosis have received more attention. For instance, it is unknown if cuproptosis is necessary to activate particular Cu enzymes, the majority of which are engaged in oxygen activation and reduction. In apoptosis and ferroptosis, mitochondrial stress can result in a significant reduction of mitochondrial membrane potential. The impact of ES-Cu on the membrane potential and the dynamics of the mitochondria must be elucidated further. It is possible that activation of mitochondrial quality regulation mechanisms, including mitochondrion-specific autophagy or mitophagy, restrict cuproptosis. Nevertheless, the protein degradation machinery (such as ubiquitin-proteasome system and autophagy) modulating proteotoxic stress to regulate cuproptosis merits further investigation. Due to the possibility that some modes of cell death cause greater inflammation than others, comprehending how cuproptosis originated, disseminated, and eventually carried out could have significant implications in the development of therapeutic approaches and prospective combination therapies. UC and CD are the most prevalent forms of IBD, which is a chronic, non-specific inflammation-related intestinal disease with an unknown cause. Extreme instances may necessitate hospitalization and surgery, have a low fatality rate, and necessitate lifetime therapy. Currently, the pathogenesis of IBD is unknown. The majority of researchers believe that it may be caused by a number of factors, including genetic susceptibility, persistent intestinal infection, intestinal flora disorder, changes in intestinal mucosal permeability, environmental and dietary habits, which may result in abnormal immune responses in intestinal mucosal tissues and intestinal inflammation. In recent years, an increasing number of studies on the pathophysiological pathogenesis and biomarkers of IBD have been conducted, which have important guiding value for clinical diagnosis and treatment, particularly in the diagnosis of disease, disease typing, drug selection, targeted therapy, efficacy prediction, disease status assessment, prognosis and recurrence prediction, etc. Consequently, basic and clinical translational research of IBD emphasizes its significance, which not only elucidates the disease’s pathophysiology but also advances clinically accurate diagnosis, stratified therapy, prognosis, and prognosis. However, studies on the combined impact of CRGs and their functional traits on IBD are scarce. By examining the association between CRGs and IBD and alterations in the immune microenvironment, this study contributes to a preliminary understanding of the significance of this novel cell death pattern in IBDs and identifies potential therapeutic targets and prognostic indicators.

To investigate if the CRGs’ expression affected IBD immune profiles, we extracted the expression of 10 CRGs from previous study and used ssGSEA to estimate the immune cell infiltration of patients with IBD. MTF1 demonstrated a substantial positive connection with virtually all of the immune cell and function subtypes, whereas PDHB, PDHA1, LIAS, FDX1, DLD, and DLAT had an almost negative link with each of these. The findings suggested that the presence of cuproptosis in cases of IBD would be a more accurate indicator. The top 3 genes (PDHA1, DLD, and FDX1) were chosen for further clustering analysis after the correlation mean value and median value of P values of all immune score related to each gene were obtained. Then the PCA indicated distinct clustering, with Cluster 1 exhibiting low DLD and PDHA1 expression and high FDX1 expression. Notably, IBD tissues had lower levels of these three regulators than normal tissues. The primary idea behind an Alignment diagram, also known as a Nomogram diagram, is to create a multi-factor regression model (such as the widely used Cox regression, Logistic regression, and so on) and calculate the contribution of each influencing component to outcome variables (the size of regression coefficient). Each value level of each influencing component is assigned a value, and the final score is calculated by adding each score. In the final step, the predicted value of the particular outcome event is calculated by making use of the function conversion relationship that exists between the overall score and the likelihood of the result event. Both clinical practise and medical research have significantly increased their interest in this field as well as their use in it. On the basis of the substantial information that we found that differentiates illnesses, we made an effort to develop a nomogram model that can diagnose IBD. The calibration curves suggested that the outcomes that the model had predicted were, to a large extent, consistent with the results that were actually obtained. The clinical utility of the model to predict the incidence likelihood of IBD was more than that of a single independent predictive factor, which can be proven by the ROC and DCA curve analysis of the column line graph model. According to the findings of these research, it would appear that the aetiology of IBD involves all of the key CRGs.

Another cause for concern is the immunological infiltration that occurs in the intestines in IBD. In IBD, excessive cytokine storms and immune cell infiltration are believed to prevent inflammation from resolving and contribute to the recurrence of the disease as well as tissue damage. The immune response that is mediated by type 1 T helper cells (Th1) and Th17 has been linked to CD, which is a chronic inflammatory disease. On the other hand, abnormal Th2 responses have been linked to the development of UC, and the eventual imbalance of interactions with other T-cell groups (such as Treg and Th9) contributes to the complexity of IBD immunopathogenesis (33, 34). Hence, immune-related genes and subtypes were compared between the clusters. Cluster 1 was composed of more abundant activated NK cells, and regulatory T cells. Meanwhile, Cluster 2 comprised more abundant memory B cells, CD4+ T memory activated cells, and follicular helper T cells. Moreover, most critical immune-related molecules (CD44, CD276, CTLA4 and ICOS) were significantly upregulated in Cluster 2 in comparison with Cluster 1. According to the data we gathered, CD44 and T cells immunity are worthy of discussion. The critical function in gastrointestinal inflammation is attributed to CD44, which is expressed in monocytes and lymphocytes (35). Leukocyte CD44 interplays with hyaluronate in the extracellular matrix following its exit from the blood and passage through the endothelial barrier. Hyaluronate is also involved in the extracellular matrix’s organization and is enhanced during inflammation (36, 37). Moreover, signals crucial for T cell activation and suppression are transmitted by the CD80/CD86:CD28/CD152 costimulatory pathways (38). Notably, the key antigen-presenting cells in the gut are macrophages and epithelial cells. Remarkably higher levels of CD80 and CD86 costimulatory molecules are expressed by the macrophages from the IBD colon (39). In colitis murine models, the CD28-CD80 interaction predominantly contributes to overcoming the tolerance and triggering the T cells-mediated immune response. Thus, the blockade of the CD80-costimulatory axis may be a promising approach in IBD therapy (40). Our findings did not find significant differences in these costimulatory molecules between the two clusters, suggesting that this may be a common feature of IBD pathogenesis.

To further elucidate the internal impacting variables between the two clusters, screening was done for DEGs and used WGCNA for identifying the module strongly associated with clustering and IBD. In total 14 modules were determined. The greenyellow and lightcyan MEs were significantly linked (>0.5) with the clusters, whereas the greenyellow, lightcyan, and lightgreen modules had a significant association with IBD. Further analysis demonstrated that the clustering and greenyellow module had a strong relationship. The PPI network was built so that we could have a comprehensive understanding of the hub genes that the greenyellow module uncovered. In addition, the MCODE algorithm divided the network into its three primary MCODEs, which are referred to as MCODE1, MCODE2 and MCODE 3, respectively. The PLCE1, PLCD3, PLCB4, and MTM1 proteins were clustered into MCODE1 and play a role in the lipid and mitochondrial metabolic program. PLCB4 is an amplification- or polysomy-derived copy gain-driven oncogenic lipid-catabolizing enzyme that encodes the ß4 variant of phosphoinositide-specific phospholipase C isoenzymes. It can also be upregulated by increased YAP1 (41). MTM1, the founding member of the myotubularin family of PI 3-phosphatases, plays a crucial role in the endosomal PI (3)P homeostasis. Defects in mitochondrial morphogenesis, endoplasmic reticulum shape, and cellular ATP depletion caused by MTM1 dysfunction can explain myofiber hypotrophy and defective sarcoplasmic reticulum organisation in human patients who frequently appear malnourished (42, 43). MCODE2 comprised TFCP2L1, LEFTY1, FOXA2, and KLF4, which are involved in cell maturation and development. Mammary progenitor cells are responsible for producing LEFTY1, a secreted regulator of NODAL/SMAD2 signaling. At the same time, LEFTY1 suppresses SMAD2 and SMAD5 signaling, which in turn promotes the long-term proliferation of both normal and malignant epithelial cells (44). It has been found that FOXA2 plays a major role in many stages of mammalian development, including the regulation of gene expression throughout the genome in the intestinal epithelium, the formation of the node and notochord, and more. Due to significant defects in gastrulation, neural tube patterning, and gut morphogenesis caused by the lack of FOXA2, embryonic lethality results. FOXA2 is necessary for the regulation of gene expression across the genome in the intestinal epithelium and the formation of the node and notochord (45). And since it is known that differentiated intestinal epithelial cells express KLF4, this indicates that KLF4 may play a role in the transition from proliferative to differentiated states in epithelial cells (46). Additionally, MCODE3 (CFTR, CA2, SLC9A2, and CA4) was strongly correlated with the regulation of peptide and ion transport. CFTR is a crucial ion transporter for Cl^−^ and HCO3^−^ in epithelial cells and is crucial for preserving the body’s interior environment’s homeostasis. High rates of cancer and cystic fibrosis were brought on by its variations (47). Intestinal epithelial cells are where the majority of the expression of the SLC9A2 gene occurs. As was demonstrated for CFTR in intestinal stem cells, it is more probable that the intracellular pH, which is regulated by SLC9A2 in the apical membrane, is the critical factor by which SLC9A2 influences signal transduction (48). The evidence presented above from the scientific literature, along with the findings from our bioinformatics analysis, point to the possibility that the regulation of mitochondrial metabolism is connected to the development and physiopathologic mechanism of IBD. This is an important topic that needs to be investigated further in the future.

In addition, we utilised GSE36807 and GSE10616 so that we could validate the clustering that was based on CRGs. The PCA plot demonstrated that the clustering that was discussed earlier had a high degree of effectiveness in distinguishing across groups. And Cluster 1 and Cluster 2 displayed unique immunological profiles. Immunological infiltration was predicted using public databases, therefore these data should be analysed carefully to avoid misleading conclusions. In order to determine whether or not the difference has any real-world implications, additional experimental verification is required. The use of scRNA-seq profiling affords the opportunity to investigate tissues that contain multicellular components. In our study, the cells were originally extensively categorised by cell-type markers into three major compartments: epithelial cells, stromal cells, and immune cells. The intestinal epithelium is the largest mucosal surface in the human body. It acts as a barrier, both physically and biochemically, between the luminal contents and the immune system that lies beneath it. Intestinal epithelium is responsible for a variety of tasks, including the absorption of nutrients, the maintenance of a physical barrier, and the response to signals from the gut microbiota and the immune system. During the course of the past few decades, there has been tremendous progress made in the immunological mechanisms that underlie IBD. This has made it possible for new IBD treatment options to be developed. On the other hand, the cuproptosis-related mechanisms that are essential to the pathogenesis of IBD are not yet fully understood. The CRGs (PDHA1, DLD, FDX1) were highly expressed in Epithelial Cycling cells, Stem cells OLFM4 LGR5, Stem cells OLFM4 PCNA from Epithelial cells, which indicated they may play an important role in the regulation of epithelial cells regeneration. Compared to other hub genes, FDX1 was strongly expressed in monocytes (CHI3L1, CYP27A1), indicating that this gene may play a crucial role in the activation and recruitment of monocytes. For stromal cells, we can find these three genes mainly located in the Inflammatory fibroblasts (IL11, CHI3L1), Myofibroblasts (GREM1, GREM2) and Pericytes cells. In addition, we investigated the relative expression discrepancies that occurred between these CRGs in a variety of different environments. We are able to find that these genes have specific differences in different cell subtypes and under different conditions. This illustrates the heterogeneity that exists within each cell subtype as well as the specific expression patterns that these genes have in different cell subtypes. Hence, several cell clusters might need to be investigated further in next research on the colon in IBD.

There are several limitations to this study. First, owing to experimental circumstances and institution size, sufficient prospective IBD cohorts, and real-world data were lacking to support the prognostic role and stratification performance of the CRGs. Second, the potential cuproptosis-related biomarkers identified in this study require more support from the literature and laboratory confirmation. Third, the CRGs came from a public database that constantly gets updated. More genes need to be found, and it is impossible to fully evaluate the different subclusters of IBD because there isn’t enough information about important clinical factors like sex, age, disease stage, response to medication, smoking, complications, and previous treatments. Additionally, the mRNA expression values were normalized using various standard methods, which could produce divergent results. Due to the inadequate understanding of cuproptosis, the majority of current evidence is limited to the investigation of changes in gene expression and must be validated by additional functional and mechanistic research.

In conclusion, our research is the first of its kind since it combines scRNA-seq profiles with microarray samples to evaluate the expression of CRGs in IBD while also studying the association between the expression of CRGs and immune infiltration. We were able to show that the classification of IBD into two distinct groups was helped by three CRGs that were screened: PDHA1, DLD, and FDX1. Moreover, there were distinctions between the two subclusters in the immune cell landscape. The CRGs expression patterns in the relevant cell clusters were also clarified by scRNA-seq profiles, which showed that the fraction of the hub CRGs in differentiated intestinal cell clusters varied throughout IBD. As a result, the thorough reflection of IBD’s cuproptosis-related signatures that we uncovered might expand our understanding of molecular mechanisms and assist future IBD researchers with novel diagnostic hints, extra potential biomarkers, or therapeutic options.

## Data availability statement

The original contributions presented in the study are included in the article/**Supplementary Material**. Further inquiries can be directed to the corresponding authors.

## Ethics statement

In compliance with regional legislation and institutional regulations, ethical approval was not needed for this research that involved human participants. According to the national legislation and the institutional guidelines, written informed consent from the patients/participants or patients/participants’ legal guardians/next of kin was not needed.

## Author contributions

Conceptualization: LL and LPL. Methodology: LL and LPL. Software and data analysis: LPL and CHY. Validation: LL and CHY. Writing, editing, and review: LL, and CHY. Supervision:YC. All authors contributed to the article and approved the submitted version.

## Supplementary material

Supplementary Figure 1 Investigation of soft-thresholding power in WGCNA. **(A)** Clustering dendrogram of 194 samples in the GSE75214 dataset. **(B)** Heatmap showing the immune cells’ relative proportion in CD, UC, and normal tissues in the GSE75214 dataset. **(C)** For k = 2, the consensus clustering matrix. **(D)** Theoutcomes of PCA of clustering.

Supplementary Figure 2 Identification of key modules genes in two clusters. **(A)** The scale-free fit index for multiple soft-thresholding powers b. **(B)** The mean connectivity for multiple soft-thresholding powers. **(C)** The volcano plot depicting the DEG expression levels in different consensus clusters. **(D)** The heatmap showing the DEGs in different consensus clusters.

Supplementary Figure 3 Construction of a PPI network. Whole Chosen genes for the constructionof the PPI network.
