# Supplementary figures and images for: Machine learning-based solution reveals cuproptosis features in inflammatory bowel disease
Source: Front Immunol. 2023 May 18;14:1136991. doi: 10.3389/fimmu.2023.1136991 (PMC10233155; doi:10.3389/fimmu.2023.1136991)

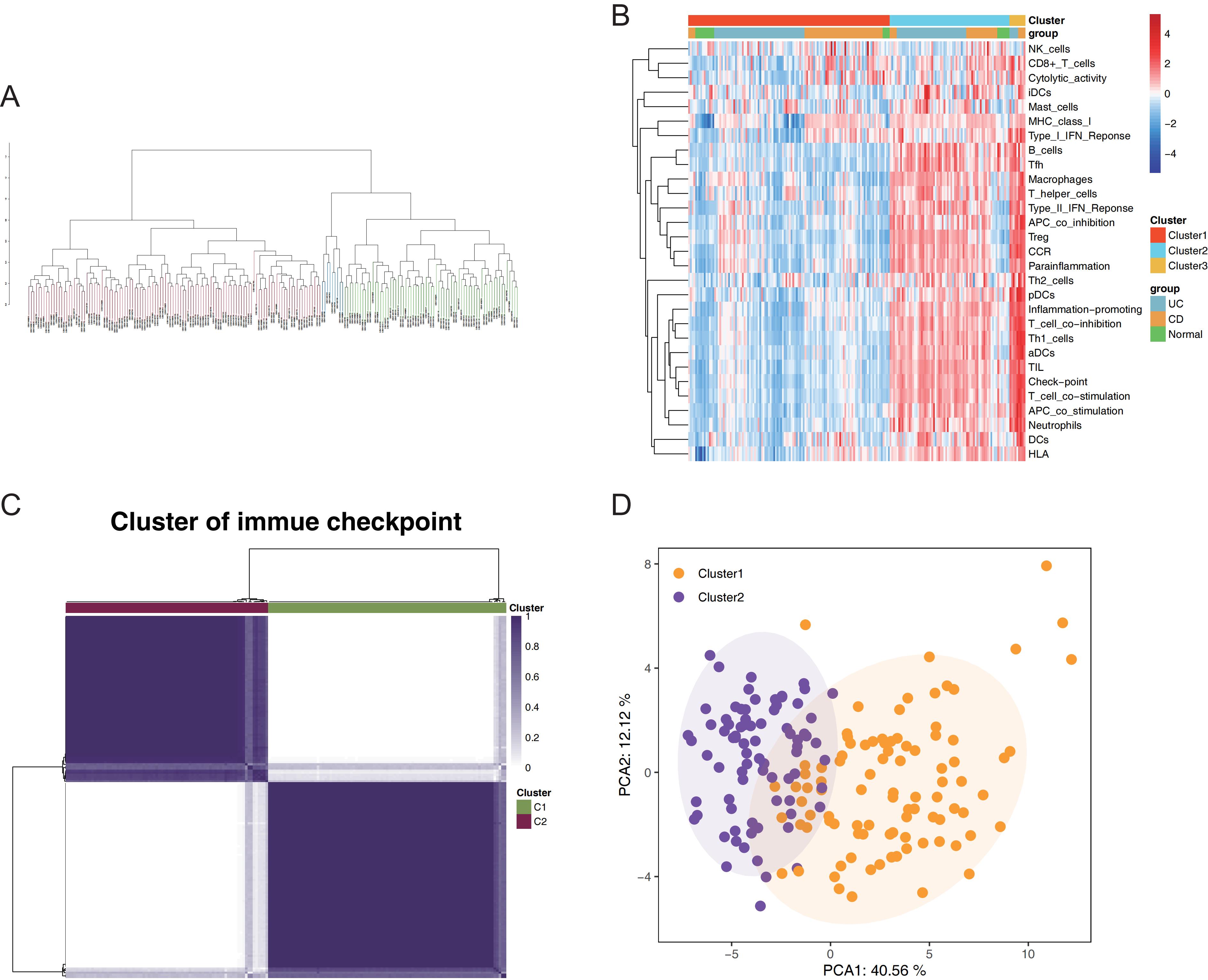

Supplement: Supplementary Figure 1 — Investigation of soft-thresholding power in WGCNA. (A) Clustering dendrogram of 194 samples in the GSE75214 dataset. (B) Heatmap showing the immune cells’ relative proportion in CD, UC, and normal tissues in the GSE75214 dataset. (C) For k = 2, the consensus clustering matrix. (D) Theoutcomes of PCA of clustering. [file Image_1.jpeg]

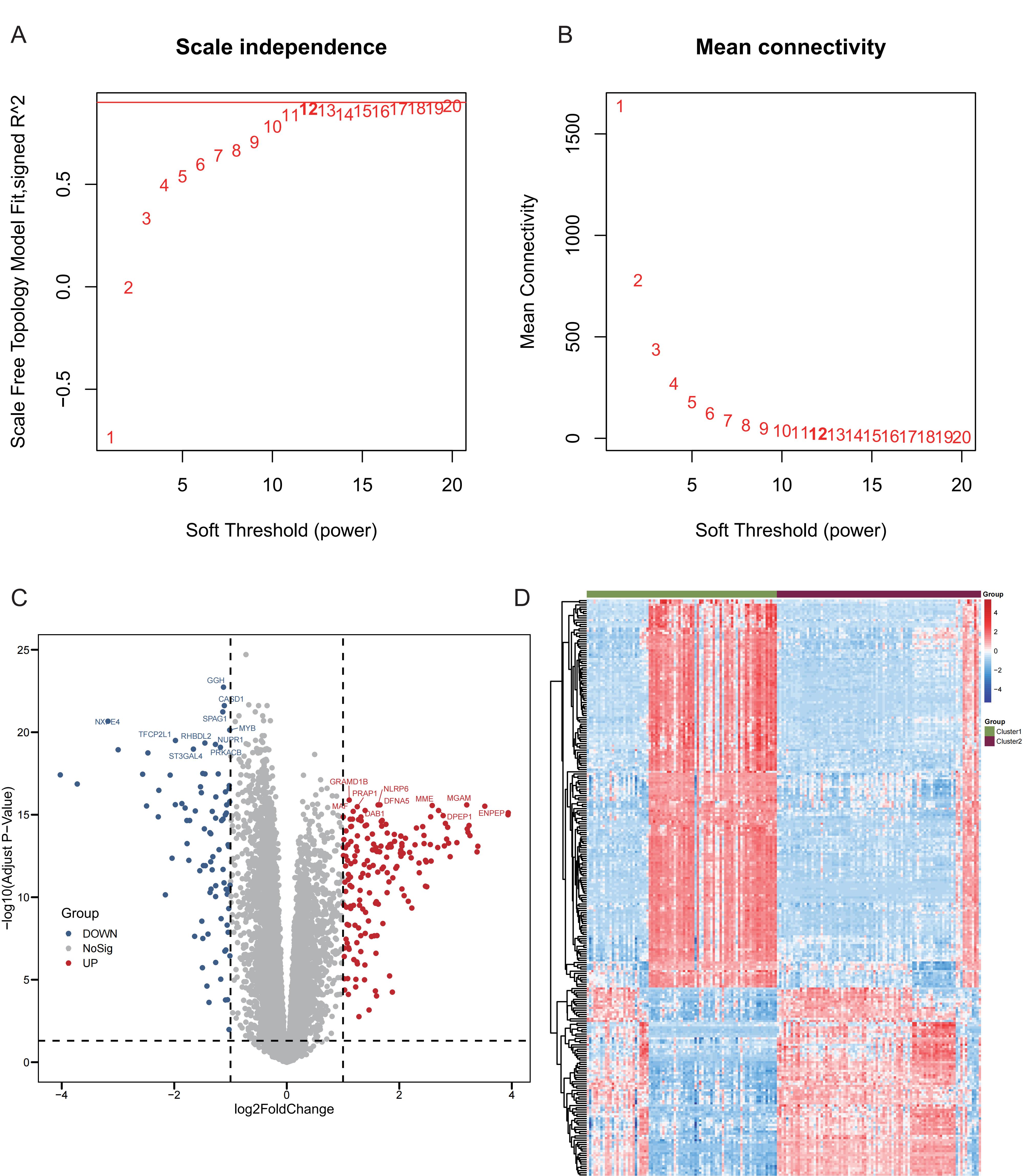

Supplement: Supplementary Figure 2 — Identification of key modules genes in two clusters. (A) The scale-free fit index for multiple soft-thresholding powers b. (B) The mean connectivity for multiple soft-thresholding powers. (C) The volcano plot depicting the DEG expression levels in different consensus clusters. (D) The heatmap showing the DEGs in different consensus clusters. [file Image_2.jpeg]

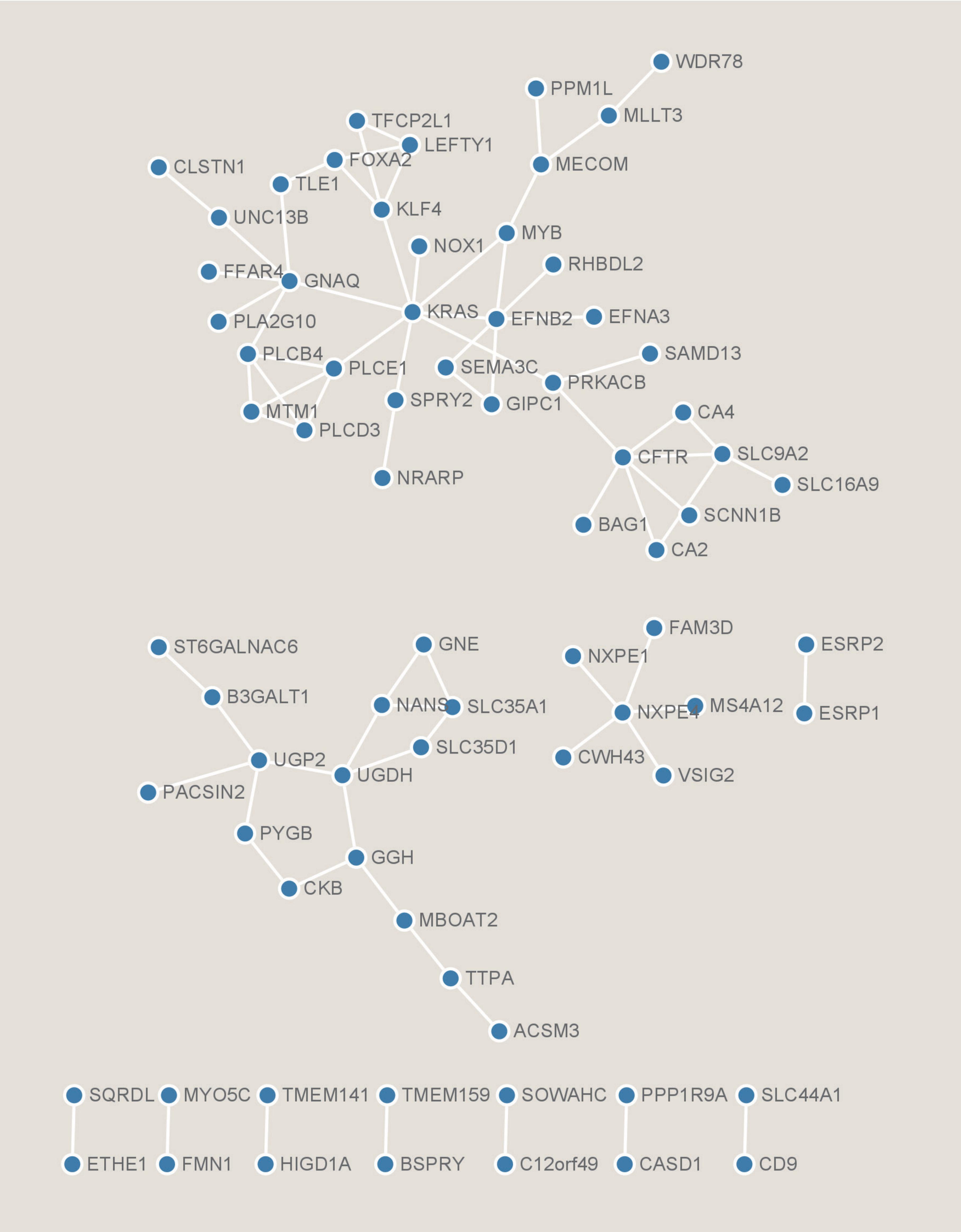

Supplement: Supplementary Figure 3 — Construction of a PPI network. Whole Chosen genes for the constructionof the PPI network. [file Image_3.jpeg]
